# Feeding behavior and trophic interaction of three shark species in the Galapagos Marine Reserve

**DOI:** 10.7717/peerj.4818

**Published:** 2018-05-25

**Authors:** Diego Páez-Rosas, Paul Insuasti-Zarate, Marjorie Riofrío-Lazo, Felipe Galván-Magaña

**Affiliations:** 1 Galapagos Science Center, Universidad San Francisco de Quito, Galápagos, Ecuador; 2 Unidad Técnica San Cristóbal, Dirección del Parque Nacional Galápagos, Galápagos, Ecuador; 3 Programa de Maestría en Manejo Sustentable de Biorecursos y Medio Ambiente, Universidad de Guayaquil, Guayaquil, Ecuador; 4 Centro Interdisciplinario de Ciencias Marinas, Instituto Politécnico Nacional, La Paz, México

**Keywords:** Feeding behavior, Stable isotopes, Trophic niche breadth, Galapagos islands, Sharks

## Abstract

There is great concern about the future of sharks in Ecuador because of the lack of biological knowledge of most species that inhabit the region. This paper analyzes the feeding behavior of the pelagic thresher shark (*Alopias pelagicus*), the blue shark (*Prionace glauca*) and the silky shark (*Carcharhinus falciformis*) through the use of stable isotopes of carbon and nitrogen (*δ*^13^C and *δ*^15^N), with the aim of determining the degree of interaction between these species in the Galapagos Marine Reserve. No interspecific differences were found in use of oceanic vs. inshore feeding areas (*δ*^13^C: Kruskal–Wallis test, *p* = 0.09). The position in the hierarchy of the food web where *A. pelagicus* feeds differed from that of the other species (*δ*^15^N: Kruskal–Wallis test, *p* = 0.01). There were no significant differences in *δ*^13^C and *δ*^15^N values between males and females of the three species (Student’s *t*-test, *p* > 0.05), which suggests that both sexes have a similar feeding behavior. A specialist strategy was observed in *P. glauca* (trophic niche breadth TNB = 0.69), while the other species were found to be generalist (*A. pelagicus* TNB = 1.50 and *C. falciformis* TNB = 1.09). The estimated trophic level (TL) varied between the three species. *C. falciformis* occupied the highest trophic level (TL = 4.4), making it a quaternary predator in the region. The results of this study coincide with the identified behavior in these predators in other areas of the tropical Pacific (Colombia and Mexico), and suggest a pelagic foraging strategy with differential consumption of prey between the three species. These ecological aspects can provide timely information when implementing in conservation measures for these shark species in the Tropical Pacific and Galapagos Marine Reserve.

## Introduction

Global increase in fishing effort has led to a decline of nearly 90% in oceanic fish populations, with elasmobranchs being one of the most affected groups (Stevens et al., 2000; Myers & Worm, 2003). Of the world’s 400 shark species, 40 are found in Ecuadorian waters, and 30 are caught in both commercial and artisanal fisheries (Jacquet et al., 2008; Martínez-Ortíz et al., 2011). The lack of biological knowledge supporting the regulation and conservation of these resources, has led to several species of sharks being listed as endangered or vulnerable by the IUCN (Clarke et al., 2006).

Most sharks are top predators, controlling trophic relationships and energy flows within the ecosystems they inhabit (Myers et al., 2007; Heithaus et al., 2008). These predators are typically considered as generalist consumers, and many have adopted strategies to exploit persistent and profitable resource regions (Au, 1991; Compagno, Dando & Fowler, 2005). However, the trophic interactions of elasmobranchs are sometimes difficult to determine using traditional methods (diet through stomach content or behavior using tagging and direct observations), so the use of alternative techniques such as stable isotopes become an opportunity to infer from another perpestive the trophic ecology of these species.

The analysis of stable isotopes of carbon and nitrogen (*δ*^13^C and *δ*^15^N) is based on the premise that these natural chemical tracers are retained in the tissues of consumer, allowing researchers to identify energy flows and characterize the resources use (Newsome et al., 2007; Martínez del Rio et al., 2009). This biogeochemical method provides information regarding general of habitat used by their prey: coastal/oceanic (*δ*^13^C), as well as the trophic strategies of a species (*δ*^15^N) (Boecklen et al., 2011; Kim et al., 2012). So the use of the isotopic niche makes it possible to infer about the foraging patterns over a spatial range (using *δ*^13^C), and the level and trophic breadth of a predator (using *δ*^15^N) (Boecklen et al., 2011; Kim et al., 2012). Differences in *δ*^13^C are determined by physicochemical, oceanographic and biological factors, which influence the taxonomic composition of phytoplankton, concentration of dissolved CO_2_ in primary consumers (Goericke & Fry, 1994; France, 1995), and the influence of carbon derived from benthic macrophytes in coastal zones that are ^13^C enriched compared to phytoplankton in pelagic environments (Michener & Schell, 1994). On the basis of this application, lower values of *δ*^13^C from predator and its prey are expected in offshore environments. For the nitrogen values, there is a strongly fractionated of *δ*^15^N values from prey to predator, resulting in isotopic enrichment from one trophic level to the next (DeNiro & Epstein, 1981; Post, 2002).

Polyspecific associations include different species that migrate, interact and forage together for different periods of time. It seems that one of the main reasons for forming such associations is the search for food (Au, 1991). The pelagic thresher shark (*Alopias pelagicus*), the blue shark (*Prionace glauca*) and the silky shark (*Carcharhinus falciformis*) are pelagic species that inhabit Equadorian waters and the tropical zones of several oceans (Markaida & Sosa-Nishizaki, 2010; Polo-Silva et al., 2013; Duffy et al., 2015). These species are mainly found in the epipelagic zone and are very active predators; so their abundance and distribution is determined by food availability (Compagno, Dando & Fowler, 2005; Rabehagasoa et al., 2012; Klarian et al., 2018). Several studies have shown that the pelagic thresher shark and blue sharks have a broad diet (e.g., cephalopods, crustaceans, pelagic and benthic fish), and their feeding behavior can vary depending on seasonal conditions, marine productivity and abundance of resources (Polo-Silva et al., 2013; Hernández-Aguilar et al., 2015; Klarian et al., 2018). While the silky shark is considered a piscivorous predator, consuming mainly fishes of the Scombridae family (Duffy et al., 2015; Estupiñán Montaño et al., 2017a).

The foraging success of sharks is linked to the diversity of their diet, which places limits on their behavior and is a decisive factor in determining the feeding strategy of these predators (Yunkai et al., 2014; Duffy et al., 2015). Species of similar evolutionary origin are susceptible to overlap in their trophic niches, which may eventually lead to the displacement or extinction of one or more populations (Hardin, 1960; Page, Mckenzie & Goldsworthy, 2005). For this reason, reducing the level of competition over food resources becomes a determining factor in facilitating the coexistence of these species, thus maintaining community structure (Bolnick et al., 2003; Pinaud & Weimerskirch, 2007). However, at the interspecific level, the partition of resources is more frequent and occurs in response to high trophic competition, allowing these populations to use different food resources to effectively reduce competition and facilitate the survival of individuals (Bolnick et al., 2003; Cherel et al., 2007; Páez-Rosas et al., 2012). In spite of this, understanding the conditions under which varying degrees of resource portioning could affect sharks remains relatively poorly understood.

In Ecuador, very little is known about the ecology, population structure and demography of sharks, including in the Galapagos Islands, an area that is a biodiversity hotspot where several species of sharks and other pelagic fish congregate (Hearn et al., 2010; Salinas de León et al., 2016). Therefore, as protection and conservation measures the commercial fishing and shark fishing are prohibited in the area, but illegal fishing boats enter the Galapagos Marine Reserve from the Ecuadorian coast (Jacquet et al., 2008; Carr et al., 2013).

Several studies have been conducted in recent years referent to habitat use and migratory patterns of different sharks in Galapagos (Hearn et al., 2010; Ketchum et al., 2014; Acuña Marrero et al., 2018). However, there is no information available on the role of these predators in the regional food web. This paper thus compares the isotopic niches of three commercially important shark species caught illegally in the Galapagos Marine Reserve and calculates the degree of niche overlap between them, with the aim of creating baseline knowledge about the trophic ecology of these species.

## METHODS

### Study area

**Figure 1:**
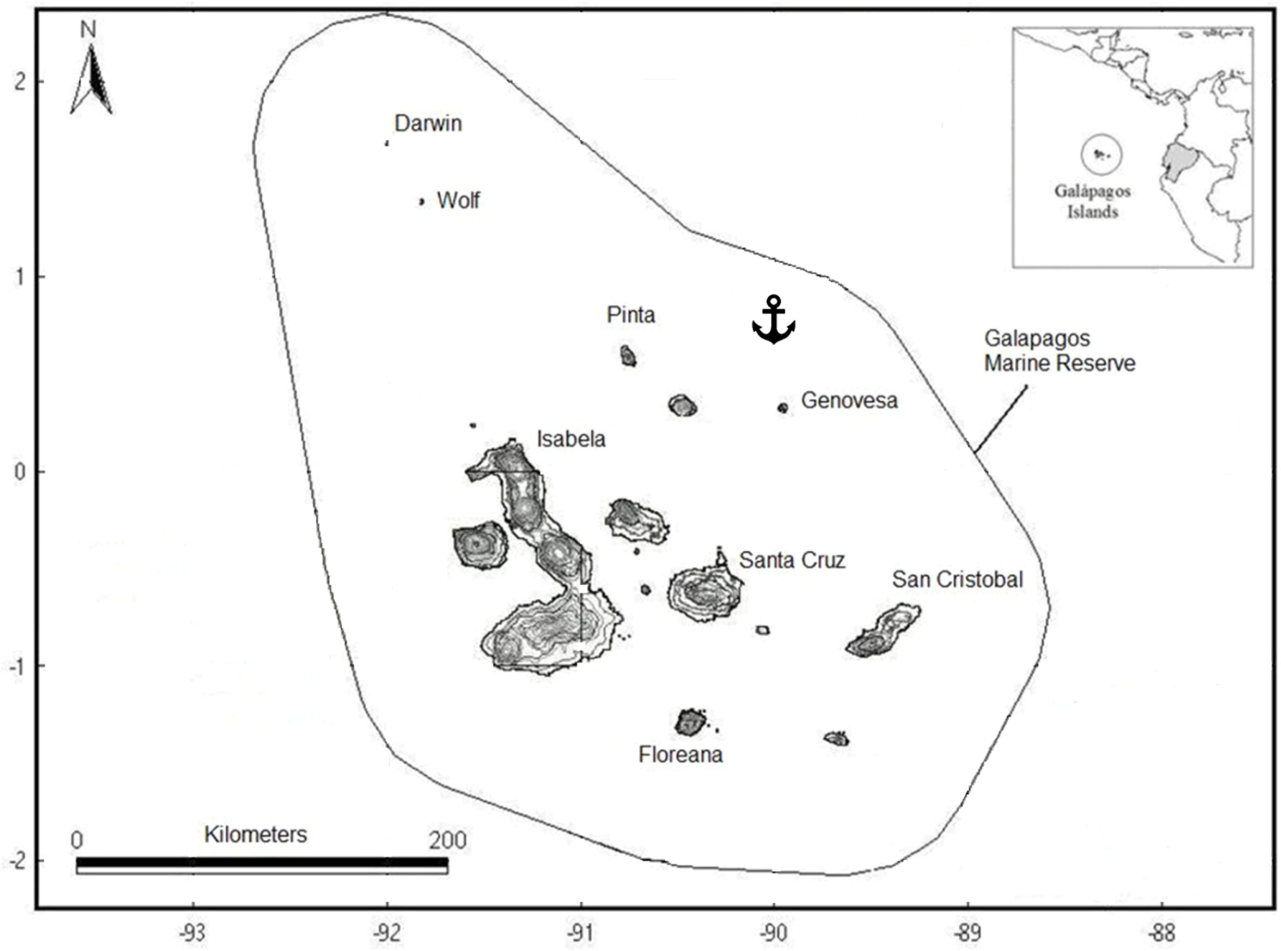
Map of the Galapagos Islands showing the boundaries of the Galapagos Marine Reserve and location where the illegal fishing boat was seized.

The Galapagos Islands are located 960 km from mainland Ecuador in the Pacific Ocean. The waters associated with this island complex form a marine reserve delineated by a “baseline” linking the outer edges of the islands to a distance of 74 km, creating a protected area of about 138,000 km^2^ (Heylings, Bensted-Smith & Altamirano, 2002) (Fig. 1). The archipelago’s unique oceanographic setting is believed to be largely responsible for the sporadic colonization of the islands, which led to the evolution of the divergent species that can be observed today in the archipelago’s ecosystems (Ryan et al., 2006; Nims et al., 2008). It is the Cromwell and Humboldt ocean currents that bring most nutrients to the region, generating, around the islands and seamounts, areas of continuous upwelling that induce phytoplankton and zooplankton blooms (Palacios et al., 2006; Schaeffer et al., 2008), hence increasing species richness and diversity in the region.

### Sample collection

This research was performed under the research permits: PC-13-12, PC-52-13 and PC-38-16; and was carried out following the protocols of ethics and animal handling approved by the Galapagos National Park and the Ecuadorian laws.

Sampling was done on board a boat seized in July 2011 by the Galapagos National Park authorities and the Ecuadorian Navy while illegally fishing sharks in the northwest region of the Galapagos Marine Reserve (0°10′18″N, 89°21′58″W) (Fig. 1). The fishing boat “Fer Mary I” from Manta, Ecuador was equipped with a 370-hook longline fishing gear and six 8-m fiberglass boats with outboard motor, to check the longline for sharks. A total of 380 sharks found in the boat’s hold were seized. For each specimen, the species and sex were identified and age group was determined based on body size. Samples of muscle tissue were taken from a total of 91 adult sharks belonging to the three species mentioned above (*A. pelagicus*, *P. glauca* or *C. falciformis*) (Table 1). All remaining shark material was then destroyed, as required by Ecuadorian laws.

**Table 1:** Values of *δ*^13^C and *δ*^1^5N in the muscle tissue of three sharks species. Total length and values of *δ*^13^C and *δ*^1^5N (expressed in ‰; mean ± SD) in the muscle tissue of three sharks species, *A. pelagicus*, *P. glauca* and *C. falciformis* in the Galapagos Marine Reserve.

**Species**	**Sex**	***n***	**Length (cm)**	*δ*^**13**^**C ± SD**	*δ*^**15**^**N ± SD**	**C/N**
*A. pelagicus*	Male	16	269.9	−16.54 ± 0.43	12.65 ± 1.58	3.08
	Female	23	271.1	−16.72 ± 0.45	12.04 ± 1.10	3.14
*P. glauca*	Male	9	174.8	−16.62 ± 0.30	13.13 ± 0.71	2.96
	Female	11	176.6	−16.65 ± 0.20	13.76 ± 1.00	2.99
*C. falciformis*	Male	20	171.7	−16.82 ± 0.30	14.26 ± 1.43	3.17
	Female	12	168.9	−16.77 ± 0.16	14.09 ± 1.49	3.05

The samples were washed with distilled water, placed into 20-ml Eppendorf tubes and then frozen to −20 °C. To confirm that all sharks were adult, the total length (TL) and precaudal length (PCL) of each specimen were measured. Total length could not be measured in *A. pelagicus*, however, because the upper lobes of caudal fins had been cut off; it was thus calculated using the allometric equations proposed by Carr et al. (2013).

### Sample processing

All muscle tissue samples were rinsed with deionized water to eliminate residues that could alter their isotopic signature, and placed in glass vials previously treated for 24 h with a chromic acid mixture prepared from sulfuric acid and potassium dichromate. They were then dried in a desiccator at 80 °C for 12 h to remove all moisture. A microwave-assisted extraction protocol (MAE) was applied (Microwave oven model: 1000-W MARS 5x, CEM, Matthews, USA) using 25 ml of a 1:1 chloroform/methanol solution (Tieszen et al., 1983) and dried again. The samples were homogenized with an agate mortar to obtain a very fine powder, of which ∼1 mg was weighed by means of an analytical microbalance with a precision of 0.001 mg and transferred into a tin capsule for isotopic analysis.

*δ*^13^C and *δ*^15^N stable isotope ratios were determined by a PDZ Europa 20–20 continuous-flow isotope-ratio mass spectrometer (Sercon Ltd., Cheshire, UK) at the Stable Isotope Facility of the University of California at Davis (CA, USA). The results, expressed in parts per thousand (‰), were obtained using the following equation: *δ*^13^C or *δ*^15^N = 1000([*R*_sample_∕*R*_standard_] − 1), where *R*_sample_ and *R*_standard_ are the ^13^C/^12^C or ^15^N/^14^N ratios of the sample and the standard, respectively. The standards used were Pee Dee Belemnite (PDB) for *δ*^13^C and atmospheric N_2_ for *δ*^15^N.

### Data analysis

Data were tested for normality and homoscedasticity using the Shapiro–Wilk and Levene test, respectively. The statistical significance of differences in *δ*^13^C and *δ*^15^N values was determined using parametric or non-parametric tests, and reported when *P* < 0.05. All statistical analyses were performed using the software Statistica 8.0.

The bayesian method SIBER (Stable Isotope Bayesian Ellipses in R) was used to define the isotopic niche space among the three species, as a measure of their isotopic resource use area at the population level. This method is based on the two-dimensional isotopic space of *δ*^13^C and *δ*^15^N, and assessed using Bayesian analysis of standard ellipses; that unlike the Euclidean methods (e.g., convex hulls), can incorporate uncertainties such as sampling biases and small sample sizes into niche metrics (Layman et al., 2007). We used Monte Carlo simulations to correct the bivariate ellipses (*δ*^13^C and *δ*^15^N), surrounding the data points in the 95% confidence interval for the distributions of both stable isotopes (Jackson et al., 2011). These corrected standard ellipse areas (SEAc) represent the isotopic niche width and the overlap parameters (Jackson et al., 2011). Furthermore, we calculated the magnitude of the isotopic overlap among the three species of sharks based on 100,000 posterior draws of the SEAc parameters (Jackson et al., 2011).

The trophic position of each shark species was estimated by the formula proposed by Post (2002): TL = *λ* + ((*δ*^15^N_predator_∕*δ*^15^N_base_)∕Δ_N_); assuming that the *δ*^15^N of a consumer and other components of the food web provides information on a species’ trophic level. The values used were the trophic position of the base species (*λ*), the *δ*^15^N value of zooplankton occurring in the region (as base), value previously reported by Páez-Rosas et al. (2012), and the isotopic fractionation factor (Δ_N_) for marine predators in general, established by Hobson et al. (1996).

## Results

### Isotopic comparison of species and sexes

**Figure 2:**
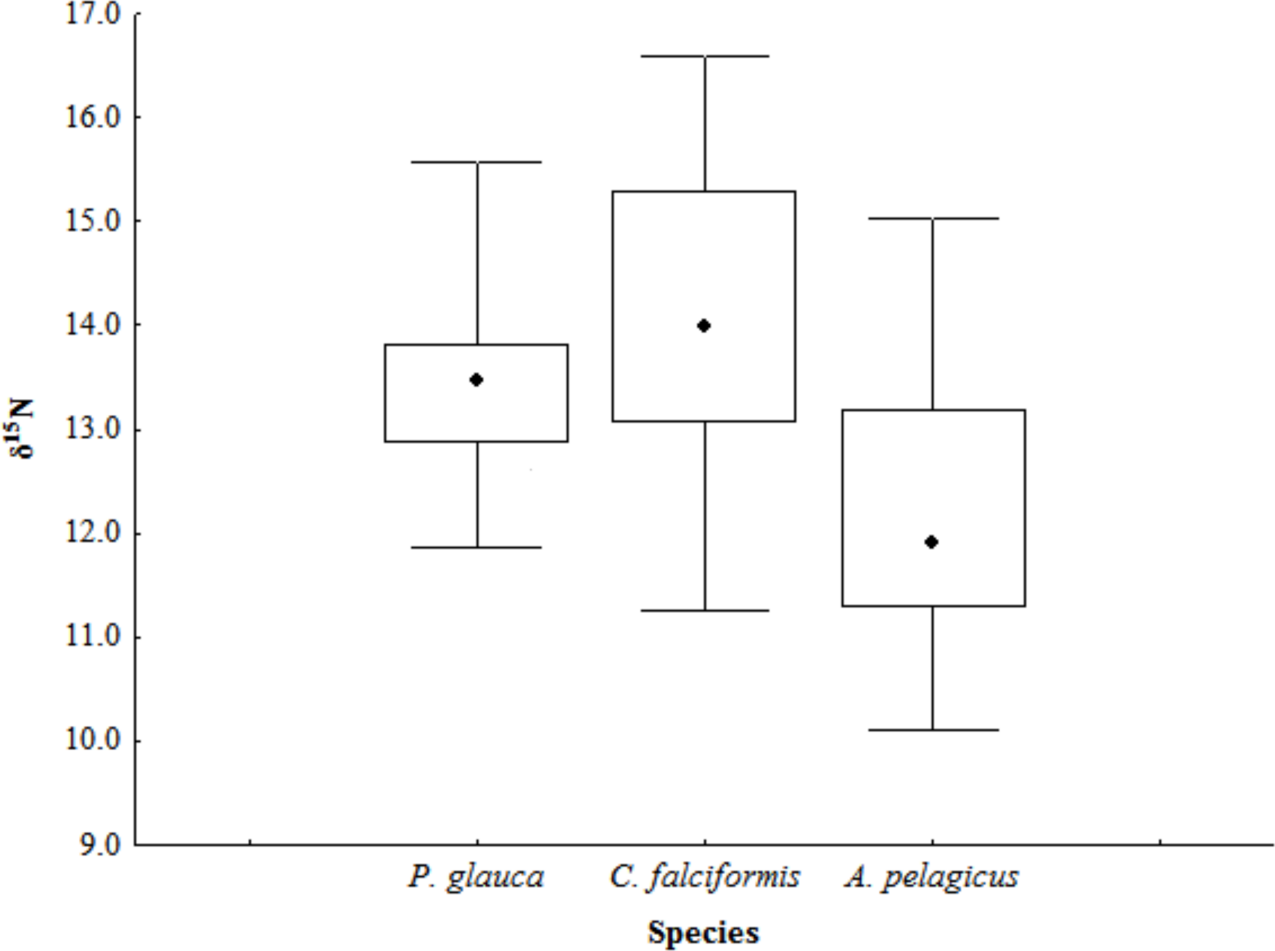
*δ*^15^N values in the muscle tissue of three shark species in the Galapagos Marine Reserve. The whiskers represents the minimum and maximum value in ‰, the black square contains the 50, 75 percentiles, and black dot is the median value.

The mean estimated *δ*^13^C and *δ*^15^N values in the muscle tissue of *A. pelagicus* were −16.59 ± 0.44‰ and 11.89 ± 1.32‰; in the tissue of *P. glauca*, −16.66 ± 0.05‰ and 13.46 ± 0.92‰; and in the tissue of *C. falciformis*, −16.82 ± 0.25‰ and 13.99 ± 1.42‰, respectively (Table 1). The C/N ratios of the samples ranged from 2.8 to 3.2, and were thus within the theoretical range established for the assimilation of protein from a predator’s diet (McConnaughey & McRoy, 1979); therefore the isotopic values reflects the diet of these predators (Table 1). There were no significant interspecific differences in *δ*^13^C values (Kruskal–Wallis test, *p* = 0.09). The *δ*^15^N values, in contrast, were significantly different between shark species (Kruskal–Wallis test, *p* = 0.01), the *δ*^15^N of *A. pelagicus* differing from those of *P. glauca* and *C. falciformis* (multiple comparisons of median ranks, *p* < 0.05) (Fig. 2).

**Figure 3:**
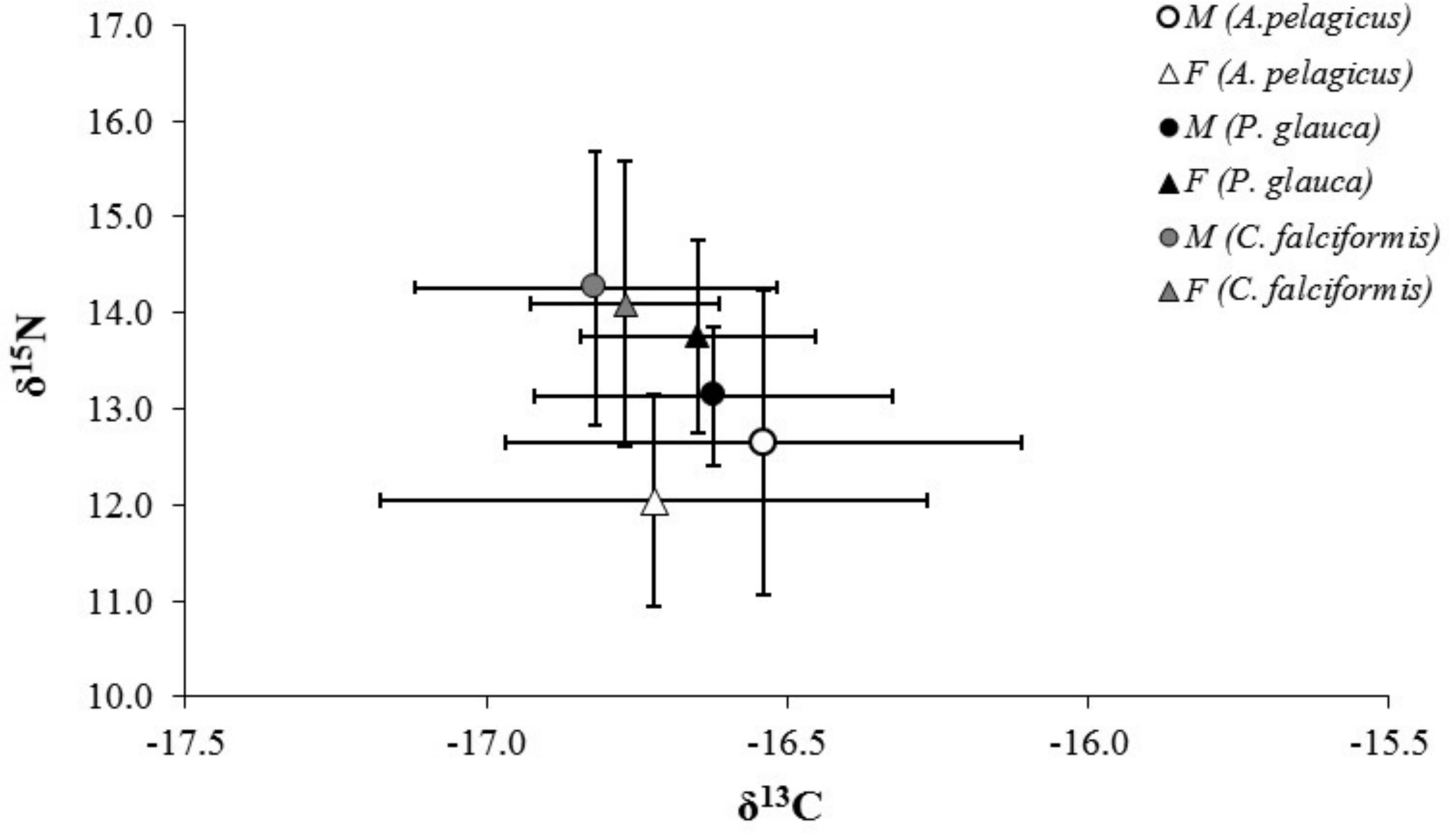
Values of *δ*^13^C and *δ*^15^N in the muscle tissue of males and females of three shark species. Values of *δ*^13^C and *δ*^15^N (expressed in ‰; mean ± SD) in the muscle tissue of males and females of *A. pelagicus*, *P. glauca* and *C. falciformis* in the Galapagos Marine Reserve.

The mean *δ*^13^C and *δ*^15^N values of *A. pelagicus*, *P. glauca* and *C. falciformis* are shown by sex in Table 1. None of the three species showed significant differences in *δ*^13^C and *δ*^15^N values between the sexes (*A. pelagicus*: Mann–Whitney *U* test, *p* = 0.22 and 0.23, respectively; *P. glauca*: paired *t*-test, *p* = 0.81 and 0.13, respectively; and *C. falciformis*: Mann–Whitney *U* test, *p* = 0.49 and 0.76, respectively) (Fig. 3). The comparison by sex of *δ*^13^C values between the species showed that the females of *P. glauca* (mean −16.65‰) had higher *δ*^13^C than those of *A. pelagicus* (−16.72‰) and *C. falciformis* (−16.77‰), but these differences were not significant (Kruskal–Wallis test, *p* = 0.09) (Table 2). The *δ*^15^N values of the females of *A. pelagicus* (12.04‰), in contrast, significantly differed from those of the females of *P. glauca* (13.76‰) and *C. falciformis* (14.09‰) (Kruskal–Wallis test, *p* = 0.01) (Table 2). With regards to males, the only significant difference in *δ*^15^N values was between *A. pelagicus* (12.65‰) and *C. falciformis* (14.26‰) (Kruskal–Wallis test, *p* = 0.01) (Table 2).

**Table 2:** *δ*^1^5N values in the muscle tissue of males and females of *A. pelagicus*, *P. glauca* and *C. falciformis* in the Galapagos Marine Reserve. Significant differences (Tukey’s HSD test, *p* < 0.05) are shown in bold.

	*A. pelagicus*	*P. glauca*	*C. falciformis*
**Females**			
*A. pelagicus*	**X**		
*P. glauca*	**0.001**	**X**	
*C. falciformis*	**0.001**	0.785	**X**
**Males**			
*A. pelagicus*	**X**		
*P. glauca*	1.000	**X**	
*C. falciformis*	**0.001**	0.211	**X**

### Trophic level, niche breadth and niche overlap

The average estimated trophic level was 3.8 for *A. pelagicus*, 4.1 for *P. glauca* and 4.4 for *C. falciformis*, making these shark species tertiary or quaternary predators in the food web.

**Figure 4:**
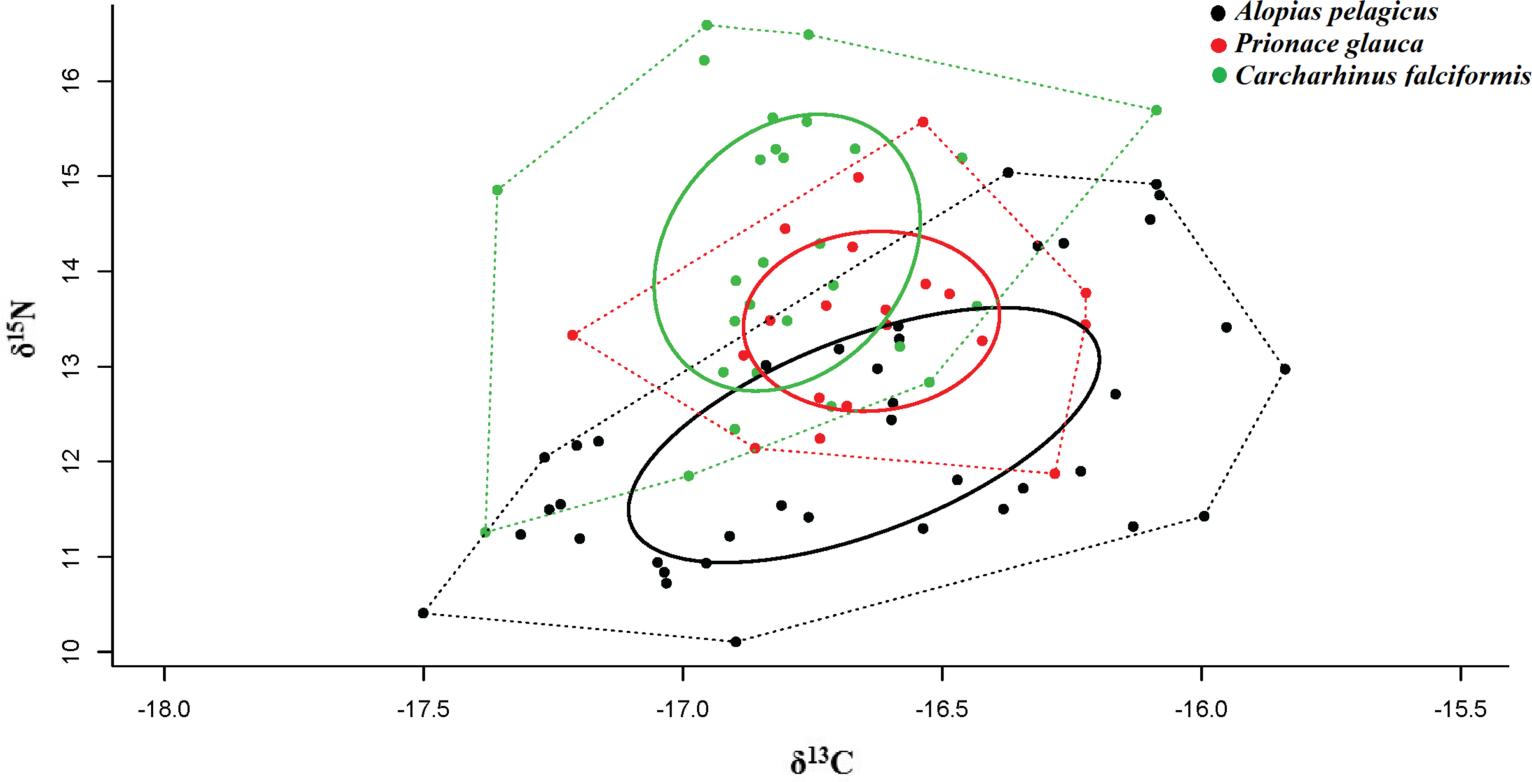
Values of *δ*^13^C and *δ*^15^N, trophic niche breadth and degree of trophic niche overlap between three shark species, *A. pelagicus*, *P. glauca* and *C. falciformis*, in the Galapagos Marine Reserve. Dotted lines represent the Convex Hull areas (polygons), while the subgroups within are formed by the standard ellipse areas corrected (SEAc), provided by SIBER analysis.

The corrected standard ellipse area (SEAc) in SIBER showed that *A. pelagicus* and *C. falciformi* s could be exploiting different types of habitat unlike *P. glauca* (Table 3). These results suggest that feeding habitat of *P. glauca* may be limited to a specific area, as opposed to the other species. The Bayesian ellipse of *C. falciformis* (SEAc = 1.14‰, 95% credibility interval of 0.82–1.46‰) and *A. pelagicus* (SEAc = 1.55‰, 95% credibility interval of 0.93–2.17‰) have a minimal overlap (Table 3 and Fig. 4), confirming different resource use patterns for these two groups of sharks. In contrast, the Bayesian ellipse of *P. glauca* (SEAc = 0.73‰, 95% credibility interval of 0.28–1.18‰) is overlapped in large part with the ellipses of the other two species (Fig. 4). The overlap area (0.33%) of the Bayesian ellipses from *C. falciformis* and *P. glauca* represented the 30.1% of the ellipse surface of the former and the 46.5% of the ellipse surface of the latter. Conversely, the overlap area (0.26%) of the Bayesian ellipses from *A. pelagicus* and *P. glauca* represented only the 19.7% of the former and the 48.3% of the ellipse surface of the *P. glauca* (Fig. 4).

**Table 3:** Basic Standard Ellipse Area (SEA) and Corrected standard ellipse area (SEAc), measured using Stable Isotope Bayesian Ellipses in R, as an estimate of the trophic niche breadth (TNB) of *A. pelagicus*, *P. glauca* and *C. falciformis*.

**Species**	**SEA**	**SEAc**	**TNB**
*A. pelagicus*	1.50	1.55	4.67
*P. glauca*	0.69	0.73	2.19
*C. falciformis*	1.09	1.14	4.19

**Figure 5:**
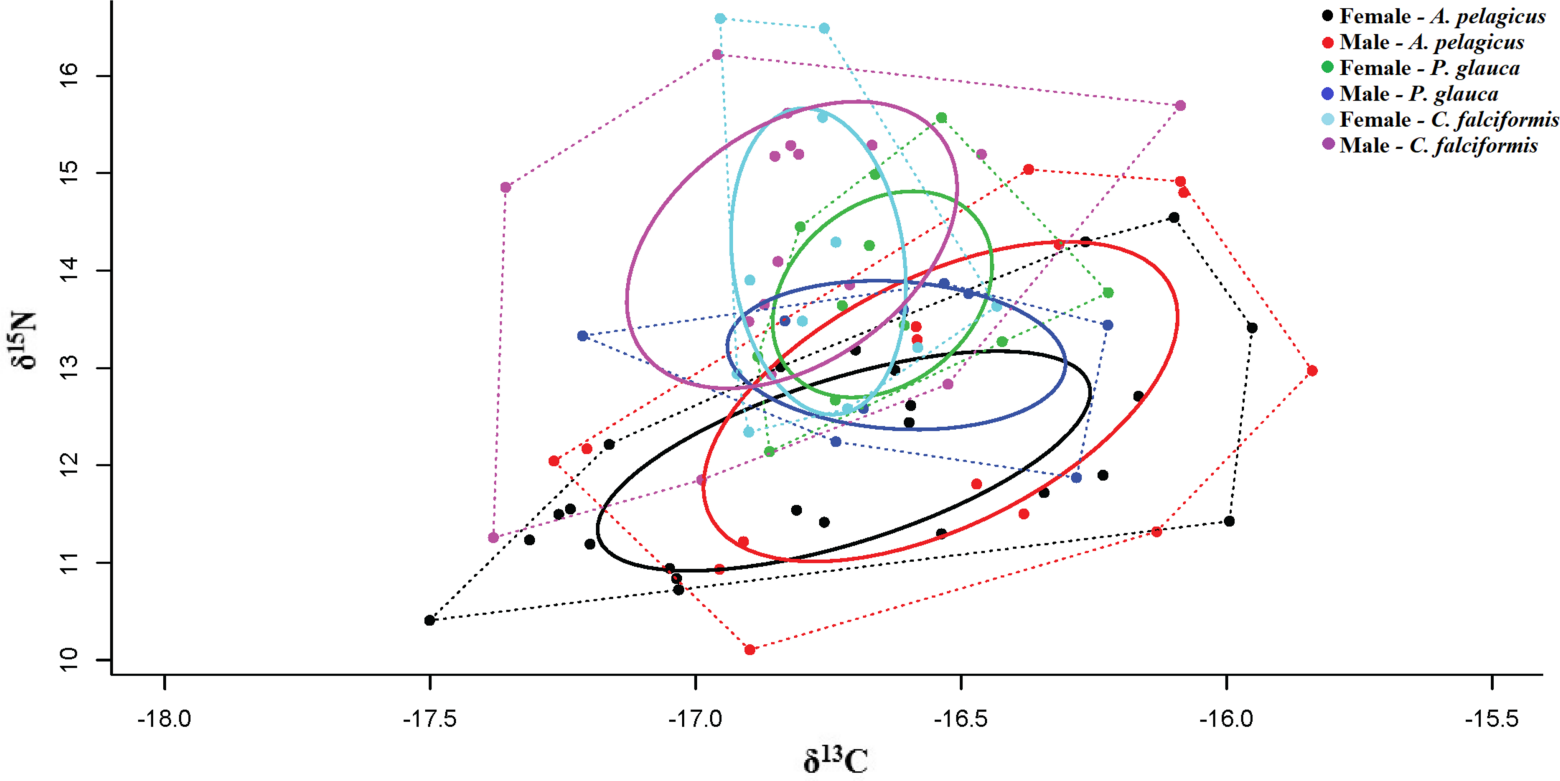
Qualitative description of the trophic niche overlap between males and females of *A. pelagicus*, *P. glauca* and *C. falciformis*. Dotted lines represent the Convex Hull areas (polygons), while the ellipse represents the isotopic niche breadth of a group of sharks.

When taking sex into account, a significant isotopic overlap was observed between males and females in *A. pelagicus* (1.00) and *C. falciformis* (0.69). While males and females of *P. glauca*, showed only a small isotopic overlap (0.36) (Table 4 and Fig. 5). In all cases, with the exception of *P. glauca*, the Bayesian ellipses of males are larger and encompass most of the Bayesian ellipses of females of the same species (Fig. 5), suggesting a higher diversity of foraging strategies in males compared to females in *A. pelagicus* and *C. falciformis*.

**Table 4:** Degree of isotopic niche overlap between males and females of *A. pelagicus*, *P. glauca* and *C. falciformis* in the Galapagos Marine Reserve. The degree of trophic niche overlap between shark populations was estimated with the overlap index of the SIBER model, where a values close to 1 (shown in bold) indicating a large overlap between their trophic niches

***Species and sex***	Female *A. pelagicus*	Male *A. pelagicus*	Female *P. glauca*	Male *P. glauca*	Female *C. falciformis*	Male *C. falciformis*
Female *A. pelagicus*	**X**					
Male *A. pelagicus*	**1.00**	**X**				
Female *P. glauca*	0.05	0.32	**X**			
Male *P. glauca*	0.30	0.36	**0.62**	**X**		
Female *C. falciformis*	0.05	0.19	0.39	0.31	**X**	
Male *C. falciformis*	0.01	0.09	0.39	0.23	**0.69**	**X**

## Discussion

### Interspecific differences

It is widely known that resource use by wildlife species may vary according to a number of factors, e.g., body size, sex or age. Depending on the availability of resources (e.g., space, food) in the ecosystem, this may lead to interspecies or within-species resource segregation (Bolnick et al., 2003; Matich, Heithaus & Layman, 2011).

In this study, no differences were observed in the foraging habitats among the three shark species. The *δ*^13^C values (between −17.2 and −16.2) indicate an oceanic/pelagic foraging strategy, consistent with what was previously reported in various studies conducted in the coasts of Ecuador, Colombia, Chile and Mexico (Polo-Silva et al., 2013; Hernández-Aguilar et al., 2015; Estupiñán Montaño et al., 2017a; Estupiñán Montaño et al., 2017b; Klarian et al., 2018). Spatial variation in *δ*^13^C values of predators can be partly explained by differences in the isotopic composition of primary producers, which are the main energy suppliers for the food web (Goericke & Fry, 1994; Pancost et al., 1997). Indeed, different levels of primary production, concentration of dissolved CO_2_, macro and microalgae composition and phytoplankton growth rate, among other factors, create a coast–ocean gradient in C-isotopic composition, with a decrease in *δ*^13^C from the coastal to the pelagic zone (France, 1995; Newsome et al., 2007).

These variability factors are present in the marine ecosystem of the Galapagos Islands which, despite being located in tropical (oligotrophic) waters, are characterized by highly productive local conditions due to the “island-mass effect” (Palacios, 2002), the combination of oceanographic currents flowing there generating high productivity around the islands (Feldman, 1986; Palacios et al., 2006). A *δ*^13^C-enriched isotopic signature, which was not observed in this study, would thus only be expected if the three shark species had foraged close to the insular shelf.

The *δ*^15^N values revealed differences in the trophic position occupied by the three shark species, *A. pelagicus* being a lower trophic-level predator compared with the other two species. The consumption of prey from different environments (epipelagic or mesopelagic) and different trophic levels is reflected by *δ*^15^N values that differ between populations exploiting similar habitats (Takai et al., 2000). These differences in *δ*^15^N values may be due not only to differential consumption of prey, but also to isotopic differences at the base of the food web (Newsome et al., 2007). Such isotopic differences manifest themselves at higher trophic links through bioaccumulation of *δ*^15^N from prey to consumer and the resulting isotopic enrichment along the food chain (Minagawa & Wada, 1984; Newsome, Clementz & Koch, 2010).

In Ecuadorian waters, *Alopias pelagicus* has been shown to feed on squid and small fish (Polo-Silva, Rendón & Galván-Magaña, 2009; Polo-Silva et al., 2013), with a preference for epipelagic squids like *Sthenoteuthis oualaniensis* (Galván-Magaña et al., 2013). The reported prevalence of these preys suggests that *A. pelagicus* forages in epipelagic waters, possibly at night when squid are more likely to be caught (Markaida & Sosa-Nishizaki, 2010). While *P. glauca* is known to be mainly teuthophagous in the North Pacific (Kubodera, Watanabe & Ichii, 2007; Hernández-Aguilar et al., 2015), recent studies carried out in South Pacific mention that its diet is based in pelagic fish, reaching even to consume marine mammals that are dead as dolphins (Klarian et al., 2018). This shark has a greater diving capacity than the other two species, allowing it to explore various habitats along the water column (Carey, Scharold & Kalmijn, 1990; Roper, Sweeney & Hochberg, 1995). It even undertakes daily vertical migrations to depths of over 600 m in order to feed on mesopelagic cephalopods occupying a high trophic level, like *Vampyroteuthis infernalis* and *Ancistrocheirus lesueurii* (Kubodera, Watanabe & Ichii, 2007; Galván-Magaña et al., 2013).

The highest *δ*^15^N values were found in *Carcharinus falciformis*, making it the highest trophic-level predator of the three species. This result agrees with other trophic studies that found this shark to have a certain preference for high-energy oceanic prey like fish of the Scombridae family (Duffy et al., 2015; Estupiñán Montaño et al., 2017b). Consistent with these references and the isotopic values observed in this study, it could be assumed that *C. falciformis* spend most of its time in surface waters at a depth of ∼50 m, conditions that are usually favorable for the aforementioned preys (Filmalter et al., 2010).

### Intersexual differences

A dietary study of populations of *A. pelagicus* occurring along the coasts of Ecuador found that their diet varies with stage of sexual maturity, but that male and female adults feed on the same prey, mostly juvenile squids of the species *D. gigas* and *S. oualaniensis* (Polo-Silva, Rendón & Galván-Magaña, 2009). Isotopic analyses of adult specimens caught by the fishing fleet at the Ecuadorian ports of Manta and Santa Rosa showed that male adults have higher *δ*^15^N values than females. Generally in most shark species the females are bigger in size compared to males, this condition allows them explore more feeding zones. While the males normally stay close the bottom to search protection and easier prey to catch (Compagno, 1990; Polo-Silva et al., 2013). These characteristics could influence that the males consume larger prey associated with the bottom, habitats that are enriched in ^15^N, so this behavior can explain this difference.

The females of *A. pelagicus* had *δ*^13^C and *δ*^15^N values that were slightly more negative and with smaller variance than those of males. This suggests that females adopt a foraging strategy that is more oceanic and focused on specific prey, while males exploit different ecosystems (oceanic–coastal), with a higher diversity of foraging strategies. Isotopic variability within a population is known to reflect, up to a certain point, dietary heterogeneity among individuals, their high mobility among feeding areas allowing them to consume different prey (Estrada et al., 2003; Jaeger et al., 2009).

The populations of *P. glauca* in the North Atlantic, is known to exhibit spatial segregation of the sexes, the males tending to frequent coastal waters more intensively than females, which are completely oceanic (Carey, Scharold & Kalmijn, 1990; Vandeperre et al., 2014). This may influence the level of consumption/abundance of their main prey (Henderson, Flannery & Dunne, 2001; Hernández-Aguilar et al., 2015). This differential behavior of males and females could explain what was observed in this study, since despite not observing statistical differences; the females had higher *δ*^15^N values than males. In light of the above studies, the spatial heterogeneity in *δ*^15^N values between sexes can be explained to some extent by the Galapagos Islands’ oceanographic setting, which allows high levels of primary productivity—not characteristic of the region—to be maintained throughout the year (Banks, 2002; Palacios et al., 2006).

With regards to the feeding behavior of *C. falciformis*, both isotopes showed no intersexual difference, which suggests that both sexes exploit similar prey and foraging habitats. Dietary studies conducted on the eastern Pacific Ocean has reported fish of the Scombridae family and squid as the species’ main prey (Duffy et al., 2015; Estupiñán Montaño et al., 2017a; Estupiñán Montaño et al., 2017b). Other authors have found males of *C. falciformis* to be more active than females, undertaking vertical migrations in the evening to complement their diet with squid and rest there at night (Compagno, Dando & Fowler, 2005). Such a behavior may explain the high variability observed here in the *δ*^13^C and *δ*^15^N values of males.

### Trophic level, niche breadth and niche overlap

One method to estimate trophic levels is to use the isotope fractionation occurring in the food chain. The formula proposed by Post (2002), used in this study, allowed *A. pelagicus* and *P. glauca* to be classified as tertiary predators, and *C. falciformis*, as a quaternary predator (Mearns et al., 1981). The trophic levels (TLs) obtained for *A. pelagicus* (TL = 3.8) and *P. glauca* (TL = 4.1) agree with the diet previously reported for both species, based mainly on small or juveniles squid and fishes of intermediate trophic levels (Polo-Silva et al., 2013; Hernández-Aguilar et al., 2015). A higher trophic level was estimated for males in *A. pelagicus*, which may be associated with them having a greater ability to approach coastal areas, where they can feed on fish that are larger and occupy a higher trophic level (Rosas-Luis et al., 2017).

The highest trophic-level predator was *C. falciformis* (TL = 4.4), which agrees with the feeding habits previously reported for this species, consisting in targeting large carnivorous fish such as tuna, black skipjack and supplement their diet with other prey like cephalopods (Duffy et al., 2015; Estupiñán Montaño et al., 2017b). Such as feeding behavior could explain the species’ heavy isotopic signature and allows to put forward the hypothesis that these sharks feed on other high trophic-level predators occurring in the region, e.g., Galapagos sea lions, which have *δ*^15^N values ∼1.5‰ lower (equivalent to prey-to-predator fractionation) than those of *C. falciformis* (Páez-Rosas & Aurioles-Gamboa, 2014). This would explain the origin of the attack marks (shark bites) that are observed in the pinnipeds of Galapagos Islands in different regions of the archipelago.

Sharks have long been classified as opportunistic predators, feeding on the resources available at a given place and time (Calow & Tytler, 1985). In recent years, it has been shown for some shark’s species that although their food spectrum includes a large variety of prey, the largest part of their diet is made up of three or four prey species, which may change with the seasons (Polo-Silva et al., 2013; Loor-Andrade et al., 2015). The trophic breadth of the three shark species was calculated based on isotopic niche breadth, resulting in a generalist type of feeding strategy for *A. pelagicus* and *C. falciformis*, and a specialist strategy for *P. glauca*. It is possible that given the great diving capacity that *P. glauca* (Roper, Sweeney & Hochberg, 1995), this species is only using a specific habitat, being more selective in the use of the resources that exist in that space.

This result about *A. pelagicus* differs from those of other studies that reported this species as a specialist predator based on stomach content analysis (Polo-Silva, Rendón & Galván-Magaña, 2009; Polo-Silva et al., 2013). Several studies mention that *A. pelagicus* shows some variability in trophic niche breadth over its life, depending on its stage of sexual maturity and the resources available in the environment (Compagno, 1990; Gerking, 1994). It is thus likely that these sharks tend towards a specialist feeding strategy as their energy requirements increase (e.g., for reproduction, gestation, etc.).

The narrower trophic niche breadth estimated for *P. glauca* suggests that this species exploits specific prey and environments, consistent with the results of Hernández-Aguilar et al. (2015) and Klarian et al. (2018) who found that populations of *P. glauca* off the coasts of Baja California and Chile show a high degree of specialization, as evidenced by the reduced number of food components that are well represented in their diet.

This study allowed *C. falciformis* to be classified as a generalist predator in the Galapagos Marine Reserve. It has been shown, however, that when there is a lower diversity of prey in the environment, these sharks tend to be more selective in choosing them (Cabrera-Chávez-Costa, Galván-Magaña & Escobar-Sánchez, 2010; Filmalter et al., 2010). Other authors have described this shark as a specialist consumer, because despite feeding on a wide food spectrum, a large proportion of its diet is made up of a limited number of prey types (Duffy et al., 2015; Estupiñán Montaño et al., 2017a). This may explain to some extent the great variability in *δ*^15^N values observed in this study.

Population-level approaches have often used trophic position and niche breadth to understand interactions within the food web and the role of predators in the ecosystem (Bearhop et al., 2004). Isotopic niche analysis showed have a minimal overlap between *A. pelagicus* and *C. falciformis*, which suggests that both species feed on prey from different trophic levels and different feeding areas (Jackson et al., 2011). In contrast, the small (but non-significant) overlap between the isotopic ellipse of *P. glauca* and those of the other two shark species reveals the consumption of a similar combination of prey and use of the same feeding areas (Matich, Heithaus & Layman, 2010). *P. glauca* would thus be more susceptible to competition for food resources (e.g., squid) and even feeding areas (Newsome et al., 2007; Graham, Spalding & Sheppard, 2010).

## Conclusions

The highly selective foraging behavior in *P. glauca*, and the existence of interespecific variation in feeding strategies of the three populations may give rise to particular energy needs, depending on the availability of the different types of prey that occur in its habitat. This would lead to some degree of specialization for certain prey, which could also be part of the food spectrum of other sharks with similar feeding strategies. Our work is the first study that uses this methodological approach to provide novel insights into the trophic ecology of sharks in the Galapagos Islands. In any case, it should be reminded that the isotopic niche is only a proxy for the trophic niche, and that the absence of significant differences between isotopic ellipses does not necessarily mean that trophic niches are identical. Further research based on stomach content analysis is thus needed to obtain more accurate information on the prey consumed by these three shark species in the Galapagos Marine Reserve.

##  Supplemental Information

10.7717/peerj.4818/supp-1 Supplemental Information 1 Isotopic database of Project Galapagos sharks

## Additional Information and Declarations

### Data Availability

The following information was supplied regarding data availability: The raw data have been uploaded as Supplemental File, and are available at http://www.datagalapagos.org.
